# Cybervictimization and Cyberbullying: The Role of Socio-Emotional Skills

**DOI:** 10.3389/fpsyt.2020.00248

**Published:** 2020-04-03

**Authors:** Nikolett Arató, András N. Zsidó, Kata Lénárd, Beatrix Lábadi

**Affiliations:** Department of Psychology, University of Pécs, Pécs, Hungary

**Keywords:** cyberbullying, cybervictimization, empathy, cognitive emotion regulation, moral disengagement

## Abstract

Social and emotional competences are considered to have a crucial role in cyberbullying as, e.g., difficulties concerning emotion regulation and empathy can characterize both cyberbullies and cybervictims. Although, the dynamics of socio-emotional processes underlying cyberbullying are still open for research, as e.g., there are contradicting results concerning the role of empathy in cybervictimization. Thus, the aim of our study was to explore the specific maladaptive emotion regulation strategies characterizing cybervictims and to clarify the role of empathy in cybervictimization. Furthermore, another goal was to explore whether moral disengagement characterizes cyberbullies in absence of empathic and adaptive emotion regulation skills. 524 students (214 males, aged 12–19 years) participated in our research. We used self-report questionnaires to measure cyberbullying perpetration and cybervictimization, adaptive and maladaptive emotion regulation strategies, moral disengagement, affective, cognitive empathy, and intention to comfort. Our main findings show that cyberbullying is associated with difficulties in socio-emotional competences. Cyberbullies and bully-victims demonstrate less empathic responsiveness and display higher moral disengagement than noncyberbullies. On the other hand cybervictims tend to use both adaptive and maladaptive emotion regulation strategies to cope with their negative emotions. In addition, cybervictims have higher cognitive and affective empathy than cyberbullies and bully-victims. Our findings confirm and extend the research on the relationship among socio-emotional skills and cyberbullying as well as cybervictimization. Moreover, our results have important implications for prevention programs targeting emotion regulation and empathy.

## Introduction

Although cyberbullying is a trending research topic, we still know little about the dynamics behind perpetration and victimization. Emerging research evidence have showed that cyberbullying can have serious physical and psychological impact, for example psychosomatic and depressive symptoms, anxiety, self-harming behavior and substance abuse (1–3). Therefore, prevention and intervention programs are needed to deal with cyberbullying behavior and these consequences (4, 5). To develop these programs, targeted research is needed to understand the individual and social processes influencing the engagement in cyberbullying.

Advances in communication technology may create specific opportunities for cyberbullying among adolescents (6, 7). Social media sites unintentionally support and maintain cyberbullying by forming groups, posting pictures and videos and commenting others’ shared content (8). Cyberbullying, by a definition, is *“an aggressive act or behavior that is carried out using electronic means by a group or individual repeatedly and over time against a victim who cannot easily defend himself or herself”* (9, p. 376.). Cyberbullying is characterized by many specific features that distinguish it from traditional bullying (4, 10). Kwan and Skoric (8) describe three unique characteristics that are different from traditional bullying: (a) there is a broader audience who can see the humiliation of the victim, (b) Internet has unlimited capacity, the abusive content is available for longer time, it can be downloaded and uploaded repeatedly, and (c) cyberbullies can be anonymous: approximately 20%–30% of cybervictims do not know the identity of the cyberbully (9, 11). Studies investigating the consequences of anonymous cyberbullying provide conflicting results: some studies (9, 11) showed that anonymity causes more severe harm on the victim. Whereas, Nocentini and colleagues (12) found contradictory evidence showing that being cybervictimized by a known person is more harmful. Besides anonymity, online disinhibition (13) also induces cyberbullying (14) through lack of face-to-face encounter and repercussions. Additionally, as socio-emotional skills have a significant role in traditional bullying, e.g. empathy (15) and moral disengagement (16), current research aims to explore whether they also affect cyberbullying involvement.

### Socio-Emotional Skills and Cyberbullying

Our current study suggests that adolescents’ socio-emotional skills contribute to engagement in cyberbullying activities. A large body of literature (17–23) confirm that lack of empathy could explain cyberbullying behavior among adolescents. Empathy helps individuals to take others’ perspective, to feel congruent but not identical vicarious emotions by witnessing another person’s experiences, emotions or suffering (24). Cyberbullies are unable to understand and feel the vicarious emotions of others (19, 22, 23). Moreover, cyberbullies not only show low empathy in the affective domain but they tend to lack the skill to take others’ perspective (17, 20). Further on, cybervictims also lack the skill of taking others’ perspective and feeling others’ emotions (21). Although, the link between cybervictimization and empathic skills seems to be more complicated. For instance, in some studies (19, 25, 26), findings show that empathy does not explain cybervictimization among adolescents. Further, other studies (20, 27, 28) suggest that cybervictims show empathic sensitivity to others’ affective states. Taken together, previous studies have showed a consensus on the lack of empathic skills characterizing cyberbullies, whereas the role of empathy in cybervictimization is unclear.

Emotion regulation also can serve as an important factor in cyberbullying. If youngsters are unable to use adaptive forms of emotion regulation strategies, the risk of engagement in cyberbullying increases (29, 30). The adaptive regulation of emotions has crucial role in successful social functioning (31), social competence (32), emotional and cognitive well-being (33), and regulation of aggression (34). Indeed, adolescents who dysregulate their negative emotions are more at risk to become cyberbullies (29). Cybervictims also show problems with regulating their emotions (30). Based on the Cyclic Process Model (35), if cybervictimized adolescents are not able to regulate the wide range of negative emotions—i.e. heightened levels of anger, depression, distress—that can be the antecedent of their tendency to become cyberbullies. Previous studies suggested that maladaptive emotion regulation explains perpetration of cyberbullying. Yet, it is not clear which of the maladaptive emotion regulatory strategies—blaming others, rumination, catastrophizing, or self-blame (36)—have a role in cyberbullying or cybervictimization.

Cyberbullies may use selective activation and disengagement of internal and moral standards—i.e. moral disengagement (37)—to avoid feelings of guilt in the lack of socio-emotional skills. Moral disengagement is a set of cognitive strategies that reconstruct cruel behavior as serving socially worthy or moral purposes (social and moral justification), exploit the contrast principle (advantageous comparison), use language to make the behavior socially acceptable (euphemistic language), reduce accountability for the behavior (displacement and diffusion of responsibility), ignore, minimize, or distort the consequences of the act (disregarding and denial of injurious effects) or blame the victim for the behavior (dehumanizing, attribution of blame) (38). Cyberbullies frequently use moral disengagement strategies to justify their aggressive online behavior (25, 39–41). Specifically, cyberbullies use diffusion of responsibility, distortion of consequences and attribution of blame to minimize the feelings of guilt and the consequences of their acts (25, 40). Additionally, both cyberbullies and bully-victims manipulate the reconstruction of their behavior to be seen as socially acceptable by using moral justification, euphemistic labeling and advantageous comparison (25). Although, most of the previous studies have used a generalized method to measure moral disengagement strategies (37), whereas they lack the use of a specified method [e.g. Cyber Bullying Moral Disengagement Scale, 39)] that measures moral disengagement in cyberbullying situations and might lead to a more specific conclusion about the role of moral disengagement in cyberbullying.

In sum, the findings from previous studies suggest a relationship between socio-emotional skills and cyberbullying (17–23, 29, 30, 35). Empathy, adaptive emotion regulation, and lack of use of moral disengagement strategies could be possible protective factors against cyberbullying behavior. However, findings for associations between socio-emotional competences and cybervictimization are less consistent. Previous studies reported contradictory findings from the no relationship to the high empathy associated to cybervictimization. Additionally, the specific maladaptive emotion regulation strategies cybervictims use are also unclear. Further research is necessary to understand whether impaired socio-emotional competence is responsible for the use of moral disengagement in cyberbullying.

### Aim of Study

The goal of our study was to analyze the role of affective and cognitive empathy, intention to comfort, specific adaptive and maladaptive emotion regulation strategies, and moral disengagement in perpetration of cyberbullying and cybervictimization. The first objective of our study was to clarify the inconsistent previous results and examine whether lack of empathic skills also characterize the cybervictims as well as cyberbullies. We hypothesized that cybervictims are unable to feel vicarious emotions and take others’ perspective. Another aim of this study was to explore the role of moral disengagement in cyberbullying and its relation to the role of empathy and emotion regulation in cyberbullying. Therefore, we hypothesized that whereas cyberbullies and bully-victims use moral disengagement to suppress the feelings of guilt, they are unable to understand their own as well as others’ emotions. A third goal of this study was to explore the specific maladaptive emotion regulation strategies that may have a predictive role in cybervictimization.

## Methods

### Participants

The participants were 524 Caucasian adolescents from one, rural and urban high school (40.84% boys, M=15.73, SD=1.30; 59.16% girls, M=15.72, SD=1.20), aged 12–19 years (M= 15.73, SD= 1.24). The choice of school and students was incidental based on accessibility. 6.9% of the students were cyberbullies, 13.5% were cybervictimized, 5.2% were bully-victims and 74.4% were outsiders. Ethical approval in conducting this study was granted from the Hungarian United Ethical Review Committee for Research in Psychology.

### Materials

We used a quantitative comparative correlational design by means of four anonymous self-administered questionnaires (For the mean scores, standard deviations and Cronbach’s alphas see **Table 1**):

**Table 1:** Descriptive statistics and Spearman correlation for the variables.

	Cyberbullying perpetration	Cybervictimization	Mean score	Std. deviation	Cronbach Alpha
(1) Cyberbullying perpetration (CVBS-S)	1	0.27**	13.54	4.08	0.83
(2) Cybervictimization (CVBS-S)	0.27**	1	23.45	8.53	0.87
(3) Affective empathy (EmQue-CA)	−0.24**	-0.01	12.21	2.65	0.66
(4) Cognitive empathy (EmQue-CA)	,−0.20**	0.16**	7.39	1.45	0.72
(5) Intention to comfort (EmQue-CA)	−0.23**	0.04	12.86	2.10	0.74
(6) Self-blame (CERQ)	−0.04	0.18**	10.41	3.51	0.81
(7)Rumination (CERQ)	−0.08	0.17**	11.62	4.00	0.83
(8) Catastrophizing (CERQ)	0.02	0.00	8.06	3.83	0.74
(9) Other blame (CERQ)	0.15**	0.02	8.53	2.89	0.75
(10) Acceptance (CERQ)	−0.02	0.17**	11.24	3.33	0.65
(11) Positive refocusing (CERQ)	−0.04	0.06	10.91	4.20	0.88
(12) Planning (CERQ)	−0.06	0.17**	13.40	3.70	0.81
(13) Positive reappraisal (CERQ)	−0.05	0.03	11.98	3.85	0.78
(14) Putting into perspective (CERQ)	−0.05	0.00	11.29	3.52	0.73
(15) Moral disengagement (CBMDS)	0.46**	0.04	13.45	4.13	0.73

**p < 0.01.

Short version of the Cyber Victim and Bullying Scale (CVBS-S, Arató et al., unpublished) is an abbreviated form of the Cyber Victim and Bullying Scale (42). The Cyber Victim and Bullying Scale measures both cyberbullying perpetration and cybervictimization with 22 items. The Scale of Cyber Bullying has three subscales: cyber verbal bullying, hiding identity and cyber forgery. The Scale of the Cyber Victim has the same three subscales reworded to measure cybervictimization. Using Item Response Theory (IRT) and confirmatory factor analysis we created a shorter adaptation for both scales, 11 items remaining in both scales designed to measure cyberbullying perpetration and cybervictimization without subscales. The participants of the adaptation procedure were 632 high school students (261 men, mean age=16.47, SD=1.50). Since this scale had not been used or validated before, confirmatory factor analyses was used to test whether the items reliably reflected cyberbullying. The results confirmed an acceptable model fit: CMIN/DF=2.66; RMSEA=0.06 (90% CI=0.05; 0.06); SRMR=0.07; TLI=0.92; CFI=0.094. Cronbach Alpha for the scale of cyberbullying perpetration was 0.83, for the scale of cybervictimization it was 0.87. Participants answered on a five-point scale (1=never, 2=rarely, 3=occasionally, 4=frequently, 5=always) to indicate how often they engaged in cyberbullying activities or became victims of it in the last one year.

The Empathy Questionnaire for Children and Adolescents (EmQue-CA, Overgaauw, Rieffe, Broekhof, Crone, & Güroglu, 2017) is a self-report measure consisting of 14 items and three scales: (1) affective empathy measuring the extent to which someone is feeling other’s distress, (2) cognitive empathy measuring the extent to which someone understands why others are in distress, (3) intention to comfort measuring the extent to which someone wants to help distressed others. The participants answered on a three-point Likert-type scale (1—not true, 2—somewhat true, 3—true) whether the empathy-related descriptions were true for them.

The Cognitive Emotion Regulation Questionnaire [CERQ, (36) trans. by Miklósi et al. (43)] consist of 36 items and has nine scales. Five scales measure adaptive emotion regulation strategies: acceptance, positive refocusing, planning, positive reappraisal and putting into perspective. An additionally four scales measure maladaptive emotion regulation strategies: self-blame, rumination, catastrophizing, and other blame. The CERQ uses a five-point Likert-type scale to measure the extent, subjects use the different emotion regulation strategies after a stressful event.

The Cyber Bullying Moral Disengagement Scale (CBMDS, Bussey et al., 2014) is a one factor scale consisting of eight items. Each item refers to cyberbullying and one item represents each of the moral disengagement mechanisms: moral justification, euphemistic language, advantageous comparison, displacement of responsibility, diffusion of responsibility, distorting consequences, attribution of blame, and dehumanizing. Participants implied on a four-point Likert-scale (1 - don’t agree, 4 - totally agree) to what extent they agreed with the statements.

### Procedure

After the school principal agreed to participate in the study, parents’ consent were asked. The students completed the questionnaires by paper-pencil during school hours supervised by teachers or research assistants.

### Statistical Analysis

We created four cyberbullying groups to test the differences between cyberbullies, cybervictims, bully-victims, and outsiders (students not involved in cyberbullying) using the mean scores and standard deviations (for the mean scores and standard deviation see **Table 1**). Students were considered cyberbullies if they scored higher than the sum of the mean and one standard deviation on cyberbullying perpetration scale of CVBS-S. Students scoring higher than the sum of the mean and one standard deviation on the cybervictimization scale of CVBS-S were considered as cybervictims. Students scoring higher than the sum of the mean and one standard deviation on both the cyberbullying perpetration and the cybervictimization scales of the CVBS-S were considered as bully-victims. Consequently, those scoring lower than the mean on both the cyberbullying perpetration and the cybervictimization scales of the CVBS-S were considered as outsiders.

Normality tests showed that the variables are normally distributed. Consequently, Pearson correlations, multivariate analyses of variance (MANOVAs) and linear regression analyses were used to test the associations among the variables. Pearson correlations were conducted to explore the relationship among cyberbullying perpetration, cybervictimization, empathy, adaptive and maladaptive cognitive emotion regulation strategies, and moral disengagement scales. Based on the correlational analyses we ran linear regression analyses. A regression analysis with stepwise extension was conducted to determine the predictors of cyberbullying perpetration with other blame, affective and cognitive empathy, intention to comfort and moral disengagement as independent variables. Another regression model with stepwise extension was tested to determine the predictors of cybervictimization with self-blame, rumination, acceptance, planning, and cognitive empathy as predictor variables. Multivariate analyses of variance (MANOVAs) with Bonferroni-corrected *post hoc* tests were performed to discover differences among the cyberbullying groups in empathy, moral disengagement and emotion regulation.

## Results

For the descriptive data, prevalence of cyberbullying and cybervictimization in gender and age groups see **Tables 1** and **2**.

**Table 2:** Descriptive data about the prevalence of cyberbullying and cybervictimization in gender and age groups.

	Girls (n=309) M (SD)	Boys (n=214) M (SD	12–14 years olds (n=79) M (SD)	15–16 years olds (n=309) M (SD)	17–19 years olds (n=135) M (SD)
**Cyberbullying perpetration (CVBS-S)**	12.66 (3.12)	14.80 (4.92)	13.61 (4.79)	13.33 (3.81)	13.96 (4.24)
**Cybervictimization (CVBS-S)**	22.98 (8.14)	24.14 (9.05)	21.66 (9.48)	23.22 (8.50)	24.83 (7.47)
	**Prevalence – girls (%)**	**Prevalence – boys (%)**	**Prevalence – 12–14 years olds (%)**	**Prevalence - 15–16 years olds (%)**	**Prevalence - 17–19 years olds (%)**
**Cyberbullies**	2.60	13.10	6.30	5.50	10.40
**Cybervictims**	13.90	13.10	15.20	13.90	11.90
**Bully-victims**	2.90	8.40	3.80	5.20	5.90
**Outsiders**	80.60	65.40	74.70	75.50	71.90

### Differences Among the Cyberbullying Groups (Cyberbullies, Cybervictims, Bully-Victims and Outsiders) in Empathy

The analysis of variance revealed statistically significant differences between the cyberbullying groups in affective empathy [F(3, 502)=7.78,p=0.00,η_p_^2 ^= 0.04]. According to the Bonferroni-corrected *post hoc* tests outsiders scored significantly higher than cyberbullies and bully-victims, as well as cybervictims scored significantly higher than cyberbullies and bully-victims. The two latter groups did not differ, also cybervictims and outsiders did not differ in empathy (for mean scores and standard deviations see **Table 3**). The cyberbullying groups also differed in cognitive empathy [F(3, 502)=7.14, p=0.00, η_p_^2 ^= 0.04]. Reported by the Bonferroni-corrected *post hoc* tests cybervictims scored significantly higher than cyberbullies and bully-victims. The two latter groups did not differ, as well as cybervictims and outsiders did not differ (for the mean scores and standard deviation see **Table 3**). We also found a significant group difference on the intention to comfort scale [F(3, 502)=9.35,p=0.00,η_p_^2 ^= 0.05]. According to the Bonferroni-corrected *post hoc* tests outsiders scored significantly higher than cyberbullies and bully-victims. The two latter groups did not differ. Also, cybervictims scored significantly higher than cyberbullies (for mean scores and standard deviations see **Table 3**).

**Table 3:** Results of multivariate analyses of variances (MANOVAs).

	Outsiders *(n=390)* M (SD)	Victims *(n=71)* M (SD)	Perpetrators *(n=36)* M (SD)	Bully-victims *(n=27)* M (SD)	F	df	η_p_^2^	Significant Post Hoc
Self-blame	10.21 (3.48)	11.65 (3.71)	10.03 (3.58)	10.00 (2.76)	3.66*	3, 502	0.02	V-O
Acceptance	11.16 (3.35)	11.99 (3.53)	9.97 (3.13)	11.89 (2.46)	3.31*	3, 502	0.02	V-B
Rumination	11.49 (4.01)	13.14 (3.90)	10.74 (3.95)	11.00 (3.63)	4.39**	3, 502	0.03	V-B, V-O
Positive refocusing	10.91 (4.02)	10.82 (4.69)	10.66 (4.41)	10.85 (4.38)	0.05	3, 502	0.00	–
Planning	13.25 (3.67)	14.32 (3.51)	12.03 (4.09)	13.74 (3.31)	3.40*	3, 502	0.02	V-B
Positive reappraisal	12.03 (3.82)	12.00 (3.87)	10.91 (4.11)	12.07 (3.37)	0.92	3, 502	0.01	–
Putting into perspective	11.29 (3.48)	11.45 (3.68)	10.14 (3.32)	11.63 (3.48)	1.37	3, 502	0.01	–
Catastrophizing	8.05 (3.52)	8.34 (3.24)	8.06 (2.74)	8.04 (3.39)	0.14	3, 502	0.00	–
Other blame	8.48 (2.84)	8.01 (2.45)	9.23 (3.91)	9.93 (2.83)	3.61*	3, 502	0.02	B/V-V
Affective empathy	12.40 (2.69)	12.38 (2.45)	10.80 (2.51)	10.56 (2.10)	7.78**	3, 502	0.04	V-B, V-B/V, O-B, O-B/V
Cognitive empathy	7.41 (1.37)	7.89 (1.27)	6.77 (1.80)	6.74 (1.68)	7.14**	3, 502	0.04	V-B, V-B/V
Intention to comfort	13.06 (2.04)	12.94 (1.71)	11.46 (2.59)	11.74 (2.33)	9.35**	3, 502	0.05	V-B, O-B/V, O-B
Moral disengagement	13.07 (3.74)	12.44 (4.15)	16.63 (4.32)	18.56 (4.19)	26.32**	3, 502	0.14	B-V, B-O, B/V-V, B/V-O

O, outsiders; V, victims; B, cyberbullies; B/V, bully-victims *p < 0.05, **p < 0.01.

### Differences Among the Cyberbullying Groups (Cyberbullies, Cybervictims, Bully-Victims and Outsiders) in Moral Disengagement

The analysis of variance revealed statistically significant differences among the cyberbullying groups in moral disengagement [F(3, 502)=26.32,p=0.00,η_p_^2 ^= 0.14]. According to the Bonferroni-corrected *post hoc* tests cyberbullies and bully-victims scored significantly higher than cybervictims and outsiders. The two latter groups, as well as cyberbullies and bully-victims did not differ (for the mean scores and standard deviations see **Table 3**).

### Differences Among the Cyberbullying Groups (Cyberbullies, Cybervictims, Bully-Victims, and Outsiders) in Emotion Regulation Strategies

The analysis of variance revealed statistically significant differences between the cyberbullying groups in self-blame [F(3, 502)=3.66,p=0.01,η_p_^2 ^= 0.02]. Based on the Bonferroni-corrected *post hoc* tests cybervictims scored significantly higher than outsiders. The other groups did not differ (for mean scores and standard deviations see **Table 3**). The cyberbullying groups also differed in rumination [F(3, 502)=4.39,p=0.01,η_p_^2 ^= 0.03]. According to the Bonferroni-corrected *post hoc* tests cybervictims scored significantly higher than cyberbullies and outsiders. The other groups did not differ (for mean scores and standard deviations see **Table 3**). There was also significant difference between the cyberbullying groups in other blame [F(3, 502)=3.61,p=0.01,η_p_^2 ^= 0.02]. As reported by the Bonferroni-corrected *post hoc* tests bully-victims scored significantly higher than cybervictims. The other groups did not differ in other blame (for mean scores and standard deviations see **Table 3**). The cyberbullying groups differed in acceptance [F(3, 502)=3.31,p=0.02,η_p_^2 ^= 0.02] as well. According to the Bonferroni-corrected *post hoc* tests victims scored significantly higher than cyberbullies. The other groups did not differ significantly (for mean scores and standard deviations see **Table 3**). Furthermore, there was significant difference between the cyberbullying groups in planning [F(3, 502)=3.40,p=0.02,η_p_^2 ^= 0.02]. As reported by the Bonferroni-corrected *post hoc* tests cybervictims scored significantly higher than cyberbullies. The other groups did not differ (for mean cores and standard deviations see **Table 3**).

### Determinants of Cyberbullying Perpetration and Cybervictimization

Based on the results of Pearson correlations (see **Table 1**) we conducted two linear regression analyses with stepwise extension to discover which variables could predict cyberbullying perpetration and cybervictimization. The final model of cyberbullying perpetration could account for 21% of the variability [F(5,515)=136.24,p=0.00]. Moral disengagement (Beta=0.46,p=0.00) was found to have the most influential, significant effect on cyberbullying perpetration (for detailed results see **Table 4**). Further, the final model of cybervictimization could account for 3% of the variability [F(5,512)=17.35, p=0.00]. Self-blame (Beta=0.18,p=0.00) was found to have the most influential, significant effect on cybervictimization (for detailed results see **Table 4**).

**Table 4:** Results of linear regression analyses with stepwise extension.

Linear regression models of cyberbullying perpetration						Linear regression models of cybervictimization					
Model 1	R^2^	F	df	Beta	t	Model 1	R^2^	F	df	Beta	t
Moral disengagement	0.21	136.24**	1,515	0.46**	11.67	Self-blame	0.03	17.35**	1,512	0.18**	4.15
**Model 2**	**R^2^**	**F**	**df**	**Beta**	**t**	**Model 2**	**R^2^**	**F**	**df**	**Beta**	**t**
Moral disengagement	0.02	75.64**	1,514	0.43**	10.73	Self-blame	0.02	13.15**	1,511	0.16**	3.51
Intention to comfort				−0.14**	−3.48	Cognitive empathy				0.13**	2.97
**Model 3**	**R^2^**	**F**	**df**	**Beta**	**t**	**Model 3**	**R^2^**	**F**	**df**	**Beta**	**t**
Moral disengagement	0.01	52.72*	1,513	0.41**	10.33	Self-blame	0.01	10.52*	1,510	0.12**	2.65
Intention to comfort				−0.14**	−3.55	Cognitive empathy				0.12**	2.60
Other blame				0.09*	2.36	Planning				0.10**	2.25

*p < 0.05, **p < 0.01.

## Discussion

The main goal of our study was to clarify the roles of empathy, emotion regulation and moral disengagement in cyberbullying perpetration and cybervictimization. Understanding the specific roles of socio-emotional skills can help to understand the dynamics behind cyberbullying and may serve as a base for prevention/intervention programs. Our results demonstrated a pattern of socio-emotional skills underlying cybervictimization and cyberbullying perpetration. We showed that cybervictims do not lack empathic skills. Further, they regulated their emotions in both adaptive and maladaptive ways. Moreover, moral disengagement characterized cyberbullies and bully-victims whereas they had difficulties with understanding others’ emotions and perspective.

Our first hypothesis was that cybervictims have the same problems concerning empathic skills as cyberbullies. However, our results demonstrated that cybervictims and cyberbullies differ in empathic competences. This is in line with previous findings (17, 19, 20, 22, 23) showing that cyberbullies are unable to take others’ perspective or feel vicarious emotions. In contrast, cybervictims did not show the same deficit in affective and cognitive empathy or intention to comfort. Cybervictims were more focused on others’ distress and had a stronger tendency to help others than cyberbullies and bully-victims. This result can serve as an explanation why bully-victims are engaged in cyberbullying as both perpetrators and victims. Bully-victims’ difficulties in understanding others’ emotions and perspective can be a risk factor why after cybervictimization, instead of adaptively coping with their negative experiences, bully-victims turn to cyberbullying. Whereas, cybervictims’ better empathic skills can be a protective factor against their subsequent cyberbullying perpetration. It is possible that the experience of being victimized leads adolescents to pay more attention to others’ feelings. Also, such social sensibility could be an antecedent of cybervictimization. In all, further longitudinal research could help understand more about the role of empathy. As well as empathy could serve as a base for programs against cyberbullying to help prevent cybervictims’ subsequent cyberbullying behavior and to prevent cyberbullies’ repeated aggressive acts.

Our second hypothesis was that moral disengagement plays a crucial role in cyberbullying. We showed that moral disengagement is indeed associated with cyberbullying perpetration. This is consistent with previous studies (25, 39–41) showing a link between cyberbullying and the use of moral disengagement strategies. A previous study (25) found that only cyberbullies are characterized by affective empathy deficit and heightened use of moral disengagement. In contrast, our results showed that moral disengagement characterized not only cyberbullies but also bully-victims. Cyberbullies and bully-victims used these strategies more often compared to cybervictims and outsiders. Whereas bully-victims and cyberbullies used cognitive strategies to suppress the feelings of guilt, they were unable to understand other people’s emotions and perspective. An explanation may be that cyberbullies and bully-victims disengage from moral standards in the absence of certain socio-emotional skills. They are unable to understand others’ emotions and their own affective states. Without these socio-emotional skills, cyberbullies and bully-victims will use alternative strategies to regulate their negative emotions. Further, bully-victims used other blame as an emotion regulation strategy that is also a way of moral disengagement such as attribution of blame and dehumanization. Consequently, using less moral disengagement strategies may lead to an opportunity for cyberbullies and bully-victims to learn how to understand their own and others’ emotional states.

The third aim of the current study was to find the specific emotion regulation strategies that characterize cybervictims. Our results showed that bully-victims used other blame to regulate their affective states compared to victims. According to the Cyclic Process Model (35) there is a risk of using maladaptive emotion regulation strategies for cybervictims to deal with their anger and distress. As a consequence of using maladaptive emotion regulation strategies, another risk of becoming a cyberbully emerges for cybervictims. Indeed, other blame may be the maladaptive emotion regulation strategy underlying cybervictims’ cyberbullying perpetration. Although previous results state that both cyberbullies and cybervictims are unable to adaptively regulate emotions (29, 30); our results showed specific emotion regulation strategies characterizing cybervictims but not cyberbullies. Cybervictims used a set of adaptive and maladaptive emotion regulation strategies, e.g. rumination, self-blame, acceptance and planning, compared to cyberbullies and outsiders. One possible explanation could be that cybervictims first use maladaptive emotion regulation strategies but then they switch to using adaptive ones. This shifting might be the result of their better empathic skills, or they receive social support helping them to regulate their distress adaptively. Furthermore, self-blame had a predictive role in cybervictimization. Thus, cybervictims who blame themselves for what happened to them will deem themselves victims of cyberbullying. Consequently, they will be in risk of substance use, depressive symptoms, anxiety, self-harming behavior etc. (1).

Some limitations of our study shall be noted. First, although anonymity should have lowered the risk of socially desirable answers, adolescents might have underreported their involvement in cyberbullying. On account of opportunity sampling, our sample was not representative of the Hungarian adolescent population. Further, it is important to be noted that the estimates of partial eta squared are weak, though the multivariate analysis of variance showed significant differences between the cyberbullying groups. Moreover, on account of the cross-sectional design of our study we could not test whether cybervictims regulate their emotions first by negative emotion regulation strategies and later shift to adaptive regulation. Without longitudinal data we can only hypothesize the temporal change in the use of cybervictims’ affect regulation. Also, our research did not include traditional bullying that could have been informative being highly correlated with cyberbullying. Finally, we used an unpublished scale to measure cyberbullying engagement.

Overall, our results demonstrated the importance of empathy, emotion regulation strategies and moral disengagement in both cyberbullying perpetration and cybervictimization. An interesting outcome of this study was that cybervictims used both adaptive and maladaptive emotion regulation strategies. Moreover, cybervictims were able to understand others’ emotions and perspective. Both of these results are worth further research to help understand why adolescents are victimized on the Internet and how they can be helped to adaptively overcome the consequences of cyberbullying. In addition, cyberbullies and bully-victims used moral disengagement strategies to justify their aggressive online behavior whereas they lacked empathic skills. Based on our results, decreasing the degree of using moral justification, cyberbullies and bully-victims may be capable of learning how to understand others’ and their own affective states. Consequently, our results might serve as a base for prevention/intervention programs. Higher levels of affective and cognitive empathy, intention to comfort others and adaptive emotion regulation could be protective factors against cyberbullying.

## Data Availability Statement

All datasets generated for this study are included in the article/supplementary material.

## Ethics Statement

Ethical approval in conducting this study was granted from the Hungarian United Ethical Review Committee for Research in Psychology (2017/96). The consent procedure (approved by the Hungarian United Ethical Review Committee for research in Psychology) was the following: We gave written information about the research project in the selected high schools. Whereas, our participants were aged between 14 and 18, first we asked the written consent of their parents, then the written consent of the adolescent participants. We granted anonymity for the participants and help to find psychological care if needed or asked for.

## Author Contributions

NA, BL, and KL designed the study. NA directed the research project. NA and AZ analyzed the data. All authors discussed the results and contributed to the final manuscript.

## Funding

The paper has been supported EFOP-3.6.1.-16-2016-00004 “Comprehensive Development for Implementing Smart Specialization Strategies at the University of Pécs,”17886-4/2018/FEKUTSTRAT, ÚNKP-19-3-I-PTE-156 and ÚNKP18-3-IV-PTE-135, New National Excellence Program of the Ministry of Human Capacities.

## Conflict of Interest

The authors declare that the research was conducted in the absence of any commercial or financial relationships that could be construed as a potential conflict of interest.
